# Altered Behavioural Response of Whitefly (*Bemisia tabaci*) on Tomato Associated with Biocontrol Plants

**DOI:** 10.1007/s10886-025-01649-4

**Published:** 2025-10-06

**Authors:** Cliven Njekete, Albane Noël, Samuel Matsinhe, Xavier Fernandez, Caroline Djian-Caporalino, Anne-Violette Lavoir

**Affiliations:** 1 Sophia Agrobiotech Institute, INRAE, Université Côte d’Azur, Sophia Antipolis, France; 2 LIDEA, Lescar, France; 3 Institut de chimie de Nice - ICN, Université Côte d’Azur, CNRS, Nice, France

**Keywords:** *Tagetes*, *Crotalaria*, Biocontrol plants, Repellent volatiles, Trap, Whitefly behaviour

## Abstract

The whitefly, *Bemisia tabaci*, is a significant pest in tomato production, causing extensive damage and economic losses. In pursuing sustainable pest management strategies, this study investigates the deterrent effects of *Tagetes* species (*T. erecta*, *T. patula*, and *T. minuta*) and *Crotalaria juncea* on *B. tabaci* settlement and oviposition on tomato plants. Two free dual-choice experimental setups were conducted in a climate-controlled chamber. The study confirmed the efficacy of the experimental setup, with similar *B. tabaci* dispersion and oviposition on the sides with tomato plants alone. When *Tagetes* or *C. juncea* was introduced, a significant reduction in *B. tabaci* settlement and oviposition was observed compared to the tomato control side. To identify the modes of action of the companion plants on *B. tabaci*, a follow-up experiment, modifying the spatial arrangement of the plants, was set up to discriminate between physical barrier and chemical repellent effects. The findings suggest a potential crossing between repellence and barrier effects for *Tagetes* species when *C. juncea* acted as a sinkhole, trapping the whiteflies. A DHS-ATD-GC-MS analysis revealed that the repellent effect seems more associated with the composition than the intensity of the blend. Some already known repellent volatile compounds of *Tagetes*, such as limonene, were identified, but the major ketone compounds must also be tested. This study demonstrates the effectiveness of *Tagetes* and *Crotalaria* species as biocontrol plants in pest management for tomato production. These plants reduce pest pressure and support sustainable agriculture, offering an alternative to chemical pesticides. Further research should investigate mechanisms, field applications, and broader agroecological benefits.

**Supplementary Information:**

The online version contains supplementary material available at 10.1007/s10886-025-01649-4.

## Introduction

Chemical control methods for insect pests have been increasingly questioned due to their unintended environmental impact, pest resistance development, health concerns, and restrictions on chemical uses (Desneux et al. 2007; Horowitz et al. 2011; Bass et al. 2015; Sparks and Nauen 2015; Dângelo et al. 2018; Soares et al. 2020). Consequently, there is a growing interest in sustainable pest management alternatives that explore, among others, plant diversity and its functional role in agrosystems. Biocontrol plants, as part of integrated pest management (IPM), are plants added to the agrosystems to provide pest regulation (Cook et al. 2007; Parolin et al. 2012, 2014; Parker et al. 2013; Balzan 2017; Djian-Caporalino and Lavoir 2024). They could act on pests directly (bottom-up effects) or indirectly (top-down effects) (Poveda et al. 2008; Letourneau et al. 2011; Han et al. 2022) using their insecticidal properties on pests via different mechanisms, including repellent volatile compounds, toxic compounds, attracting natural enemies, and barrier effects (Zavaleta-Mejía and Gómez 1995; Djian-Caporalino et al. 2005; Calumpang et al. 2015; Ben-Issa et al. 2017; Dardouri et al. 2017; Zuma et al. 2023).

Pest deterrence involves barrier plants and push plants that function differently but with the same goal in pest control strategies, leveraging physical and chemical mechanisms. Barrier plants create a generic physical obstruction to pest movement or feeding, relying on traits such as dense foliage, trichomes, or tough plant tissues that hinder pest access to crops, as elucidated in some studies (Fereres 2000; Hooks and Fereres 2006). Push plants emit volatile organic compounds (VOCs) that repel pests through chemical signals, e.g. as seen in garlic on aphids (Moono and Musenge 2019). A combination of VOCs (repellent/attractant) and physical barriers could potentially create a synergistic effect for pest management.

*Tagetes* spp. are probably the most well-known biocontrol plants as they are used against many pests in various contexts (Djian-Caporalino et al. 2024). From an insecticidal perspective, *T. patula* on plant assays reduced the survival of whitefly *Bemisia tabaci* more than on bean (Fabrick et al. 2020), while it repelled another whitefly, *Trialeurodes vaporariorum*, on greenhouse tomato (Conboy et al. 2019). *Tagetes erecta* reduced the incidence of unspecified whitefly species and their associated viruses in a field tomato (Zavaleta-Mejía and Gómez 1995). *Tagetes minuta* at flowering was preferred over tomato by *T. vaporariorum* in olfactometer assays (Matu et al. 2020). *Tagetes* species produce VOCs such as limonene, dihydrotagetone, (*Z*)-β-ocimene, α-pinene, (*Z*)−3-hexenyl acetate, carveol, 3-Hexen-1-ol, acetate, (*Z*)-terpinolene, linalool, and (*E*)-tagetone, which can affect herbivore pest behaviour and performance (Dardouri et al. 2017; Conboy et al. 2019; Matu et al. 2020). Thus, *Tagetes* species have insecticidal effects, but are these effects consistent across all *Tagetes* species when targeting different pests? Are the mechanisms the same, especially when the molecules may differ in type, quantity, or quality? On the other hand, *Crotalaria juncea* has been mainly noted for its potential in nematode management (Wang et al. 2002; Colegate et al. 2012; Njekete et al. 2025). Manandhar et al. (2009) demonstrated that interplanting zucchini with *C. juncea* significantly reduced silverleaf whitefly populations and the severity of squash silverleaf disorder, likely due to *C. juncea*’s role as a physical barrier to pest colonisation and its ability to enhance soil nitrogen levels, thereby improving zucchini plant vigour and resilience. In another study, *C. juncea* exhibited barrier effects against *B. tabaci* and reduced the incidence of virus transmission when used as a border biocontrol plant in pepper (Deberdt and Fernandes 2013). After all, plants with tall or large canopies are generally known to act as mechanical barriers (Fereres 2000; Djian-Caporalino and Lavoir 2024). *Crotalaria juncea* fits such criteria. However, the role of *C. juncea* in aboveground pest management, such as for whiteflies, remains understudied.

Tomato was chosen as a model crop for this study. It is a globally significant vegetable crop by cultivation and consumption (Srinivasan 2010; FAO 2023) and suffers from a plethora of pests (Galicher et al. 2017; Wakil et al. 2018; Walgenbach 2018). One of the major challenges is the management of the whitefly, *Bemisia tabaci* (Gennadius), a highly polyphagous pest capable of direct and indirect damage. Directly, it feeds and sucks sap from the plant, while indirectly it vectors plant viruses, including Tomato yellow leaf curl virus (TYLCV), which can devastate tomato yields (Oliveira et al. 2001; Horowitz et al. 2011; Navas-Castillo et al. 2011; Galicher et al. 2017; Wakil et al. 2018).

The objectives of this study were to: (1) evaluate the deterrent effects of three *Tagetes* spp. and a species of *C. juncea* on *B. tabaci* settlement and oviposition on tomato plants, (2) explore the mechanisms behind the deterrent effects by disentangling between a repellent and/or a barrier effect with two different setups, and (3) analyse the leaf VOCs emission of the *Tagetes* species to interpret the chemical repellent effect.

## Methodology

### Biological Materials

Tomato, *Solanum lycopersicum* L. cv. St. Pierre (Solanaceae) sourced from Syngenta was used as the host crop plant for *Bemisia tabaci*.

Three marigold species were tested as biocontrol plants: *Tagetes minuta* cv. unknown from Germinance, *T. patula* cv. Princess of Orange, and *T. erecta* cv. CrackerJack (Asteraceae) from Lidea. In addition, sunn hemp (*C. juncea* cv. unknown, Fabaceae) from Lidea was also tested.

All the plants were sown in small pots containing a 1:1 mixture of soil and sand in the growth chamber (16 h:8 h L: D, 23 °C ± 1 °C). They were all transferred as seedlings from the growth chamber to the greenhouse 15 days later. The plants received a nitrogen, phosphorus, and potassium inorganic fertiliser prepared at 2‰ (Nutribio 4.3.6 from Frayssinet). Tape water irrigation was done thrice a week. All the plants were used at the pre-flowering stage at 1.5 months of age.

The whitefly, *Bemisia tabaci* (Hemiptera: Aleyrodidae), was reared on tobacco plants in the climate chamber (23 °C, 16 h:8 h L: D) at the INRAE Sophia Antipolis experimental unit, after its first collection (pest strain) from the INRAE facility in Montpellier.

### Experimental Design: Free Dual-choice Assay

**Fig. 1:**
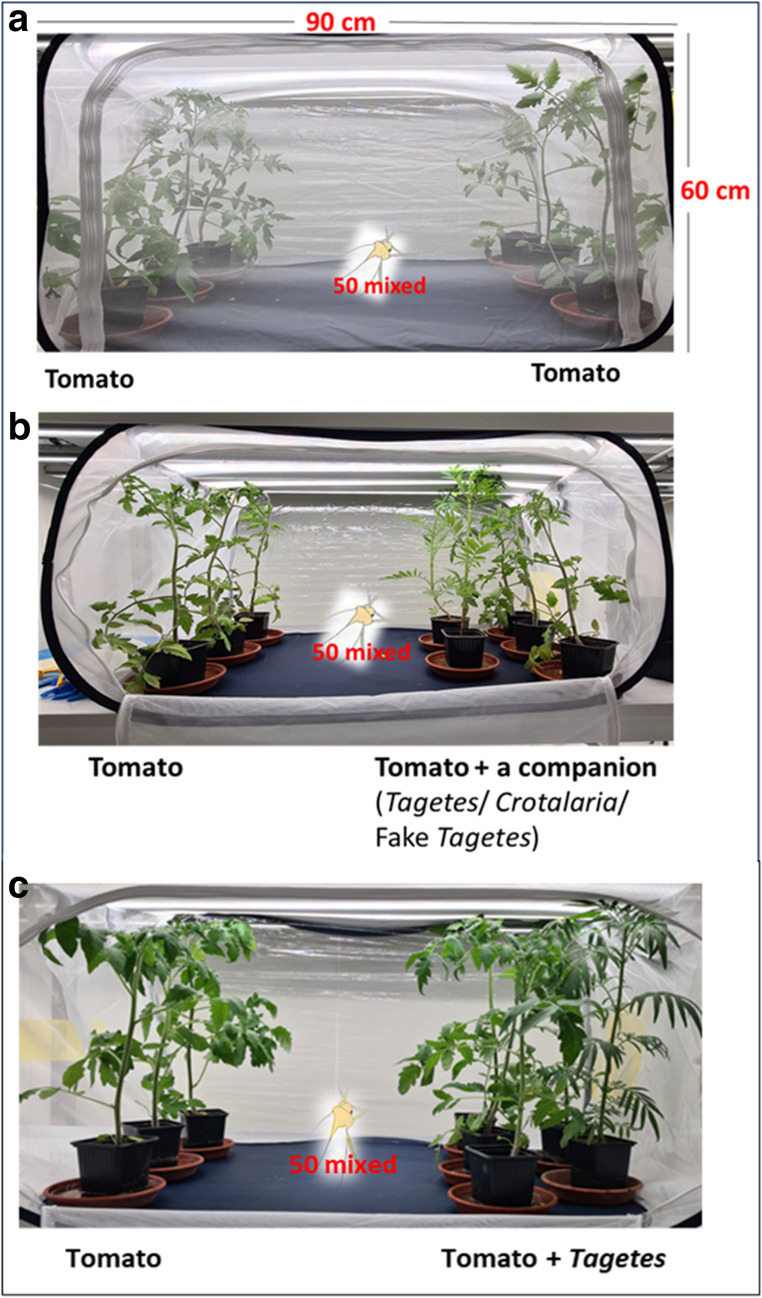
Free dual choice assay setup of plants installed in the cages. **a**. control, **b**. companion in front, **c**. companion behind. 50 mixed = 50 flying mixed-sex adults of *Bemisia tabaci*

Free dual-choice assays were performed in a climate chamber (23 °C, 70% HR, 16 h:8 h L: D, and 0042Ue light intensity). We first assessed the efficacy of our experimental setup where 50 flying mixed-sex adults of *B. tabaci* were gently inserted in the middle of a 1000 cm x 90 cm x 90 cm mesh cage, by placing a test tube containing the individuals allowing them to disperse according to their choices, where three tomato plants, approximately the same size, were arranged on the left part and the right part of the cage (Fig. 1a). This setup was repeated eight times with cages randomly installed inside the climate chamber (*n* = 8 cages). The choice of whiteflies was recorded by counting the number of adults settled on either side and every tomato plant at 24 and 48 h after release. The number of whitefly eggs was counted for each of the three locations of each tomato plant: bottom, middle, and top, after 48 h.

Then, we tested the deterrent effects of *Tagetes* species (*T. minuta*, *T. patula*, and *T. erecta*) and *C. juncea* on *B. tabaci* by using the same experimental setup, plus adding two biocontrol plants in front of one line of the tomato plants inside the cage (Fig. 1b). Biocontrol plants were added randomly in the cage on the left or the right side and the setup was repeated 8 times (*n* = 8 cages). In this setup, we strategically placed the biocontrol plants in the front to hypothetically enhance both the repellent effect and the barrier effect. Whitefly adult settlement and oviposition were counted similarly on tomato protected or not by the two biocontrol plants. After each treatment, the climate chamber was cleaned and aerated for four days to remove any volatile organic compounds that could be lingering.

To distinguish the physical barrier effect from the chemical repellent effect, the same experimental setup was repeated with a few modifications. First, to test the physical barrier effect, living biocontrol plants were replaced with fake plants in front of one range of tomato (Fig. 1b). Fake *Tagetes* plants were purchased from ArtPlants in France and had their flowers removed before use in our assays since the biocontrol plants in our experiment were used before flowering. Second, to test the repellent effect, the same living biocontrol plants were used, but were placed behind the tomato plants and not in front (Fig. 1c). This setup, which we modified from Conboy et al. (2019), has the strength to explore the repellent effect. Again, each treatment was repeated 8 times (*n* = 8 cages).

### Justification for Including Crotalaria juncea Despite Testing in One Setup

Unlike the *Tagetes*, *C. juncea* was assessed only in the ‘biocontrol in front’ setup, based on the assumption that its deterrent effect, if any, would arise from a physical barrier rather than volatile emissions. Therefore, testing it in the ‘biocontrol behind’ setup, which primarily assesses chemical repellence, and analysing the VOCs, was not pursued.

### Volatile Organic Compounds Sampling and Analysis

*VOC sampling* - Dynamic headspace sampling was used to analyse the volatile organic compounds (VOCs) emitted by the *Tagetes* species (*T. minuta*, *T. erecta*, and *T. patula*) and the fake plants (Fig. S2). Clean plants of each species were selected, and a healthy leaf from each plant was placed in a sealed inert material bag (Tedlar). The bags were tied tightly around the leaf to prevent the loss of volatiles and connected by an airflue. Headspace volatiles were collected at a 100 mL/min flow rate using a dynamic headspace flow system regulated by a flowmeter (SUPELCO, Bellefonte, PA, USA). Volatiles were trapped for 30 min, which allowed enough time for sufficient volatile extraction based on prior studies optimising flow rate and extraction time (Stierlin et al. 2020). The trap used for adsorption of VOCs consisted of stainless-steel tubes filled with Tenax^®^ TA porous polymer, 60–80 mesh, 1.6 mg (SUPELCO, Bellefonte, PA, USA), which was pre-conditioned at 300 °C for 1 h.

Three plants of each *Tagetes* species were sampled (*n* = 3). The same plastic bag was reused for each replicate of the same species to avoid cross-contamination. The three repetitions were randomised, with the species rotated each time to prevent any potential temporal bias in VOCs emissions (Fig. S2). The sampling was conducted in a greenhouse where all *Tagetes* species were grown, ensuring environmental consistency. For each replicate, an air sample (an empty bag without any leaf) was also collected to account for any background contaminants. Interestingly, these samples appeared to contain a mixture of the three *Tagetes* VOCs, likely saturating the air inside the greenhouse compartments where they were grown. We then considered this sample as a pool of VOCs circulating in the greenhouse and redid another air control sample in an empty (without plants) greenhouse compartment. Three fake plants were also sampled in the empty compartment to avoid contamination.

*VOC analyses* - After collecting the VOCs, samples were desorbed by using a 350 Automated Thermal Desorber (Perkin-Elmer, Norwalk, CT, USA). Volatile compounds were desorbed at a flow rate of 50 mL/min at 280 °C for 3 min (primary desorption) and then cryofocused on a Tenax TA trap at 5 °C. This trap was heated to 280 °C at 40 °C/sec, with the maximum temperature maintained for 5 min. The desorbed volatile compounds were transferred to the GC column through a fused-silica line heated at 280 °C in splitless mode.

The thermodesorber was connected to a gas chromatograph GC Clarus 680 coupled to MS Clarus SQ8T mass detector (Perkin-Elmer, Norwalk, CT, USA). The analyses were performed on a DB-5MS column (60 m x 0.25 mm, 1.0 μm, Agilent Technologies, Inc., USA). The oven temperature was programmed to begin at 60 °C, then to rise from 60 °C to 120 °C at 1.5 °C/min, and then from 120 °C to 250 °C at 15 °C/min and then held at 250 °C for 20 min. The carrier gas, helium, flowed at a constant speed of 1 mL/min. Mass spectra were recorded using the electronic ionisation (EI) mode at 70 eV, scanning the m/z 20–300 range.

Data acquisitions were carried out using software TurboMatrix™ and TurboMass (Perkin-Elmer). Chromatograms generated by the GC-MS were analysed using specialised software, Agilent ChemStation. Each peak, representing a specific volatile compound, was integrated, and the corresponding peak areas were recorded to quantify the relative concentrations of VOCs. Compounds which represent less than 1% of the total area intensity were not considered for further analysis.

The identification of compounds involved the comparison of mass spectra with those recorded by internal or commercial mass-spectral libraries (NIST). Retention indices (RI) were calculated using a formula described by van den Dool and Kratz, as well as according to the retention time of a standard n-alkanes C6-C18 mixture (Van den Dool and Kratz 1963). All identifications correspond to putatively identified compounds (level two) (Koelmel et al. 2022) because no standards were used in this study. The retention times of identified compounds were noted, and peak areas were quantified to determine the relative concentrations of the detected VOCs.

Pollutants found in the blank control sampling were identified and excluded from the dataset of the plant sampling for further analysis.

### Data Analysis

*Free dual-choice data* - Adult whitefly counts and egg oviposition on the tomato plants positioned on either side of the cage—alone on one side and paired with a biocontrol or fake plant on the other, for each treatment. Given this paired structure, where each whitefly could “choose” between the two sides, the data represent binomial proportions (e.g., number of whiteflies on side A vs. total number of whiteflies on both sides per replicate). Therefore, a Binomial Generalised Linear Model with a log link function was used to model the proportion of whiteflies settling on one side versus the other. The model included Treatment, Time (24 h, 48 h), and Side (tomato alone, tomato paired with a companion) as fixed factors, with an interaction term between Treatment and Side to assess differences in insect preference between the two sides for each treatment. This model is appropriate because it accounts for the binary choice outcome, allows for comparison across treatments and time points, and accommodates potential overdispersion if necessary. Post-hoc multiple comparisons were conducted using estimated marginal means (emmeans package), with *P*-values adjusted using the Sidak method to compare insect counts (a). between the two sides within each treatment and (b). among treatment groups, at both time points (24 h and 48 h). In the case of the *C. juncea* treatment, whiteflies were also observed settling directly on the biocontrol plant. To ensure fair treatment comparisons, individuals counted on *C. juncea* were excluded from the analysis. Only the counts of whiteflies settled on the tomato plants were retained, enabling consistent comparisons between tomato-alone and tomato associated with a companion, across treatments. All analyses were performed using R version 4.5.0.

*Chemical data from VOC analyses* - The volatile profiles of the three *Tagetes* species were compared to identify species-specific compounds and to evaluate similarities and differences in their emission patterns. A principal component analysis (PCA) was performed to reduce the dimensionality of the VOCs dataset and identify patterns or clusters in the volatile emissions of the different *Tagetes* species. The dataset was normalised and standardised to remove scale biases. Principal component analysis plots were generated using the first two principal components, which accounted for the majority of the variance, enabling a clear visualisation of how the species’ volatile profiles clustered.

Additionally, the total area intensity values from the GC-MS chromatographs were analysed to evaluate the semi-quantitative differences between the VOC emission profiles of the three *Tagetes* species, using a GLM with a Gamma distribution and a log link function. This approach was selected due to the continuous, positively skewed nature of the data, where the variance increased with the mean. Although our data was normal, the Gamma GLM was appropriate to address the excessive overdispersion in our data. Post-hoc comparisons between treatments were conducted using Tukey’s HSD to identify significant differences in total area intensity across treatments.

All statistical analyses were conducted using R software, with significance set at *P* < 0.05.

This combined approach allowed both qualitative and semi-quantitative assessment of the differences in VOC emission among the species, providing insight into their potential biocontrol mechanisms.

## Results

### Free-dual Choice Assay

**Fig. 2:**
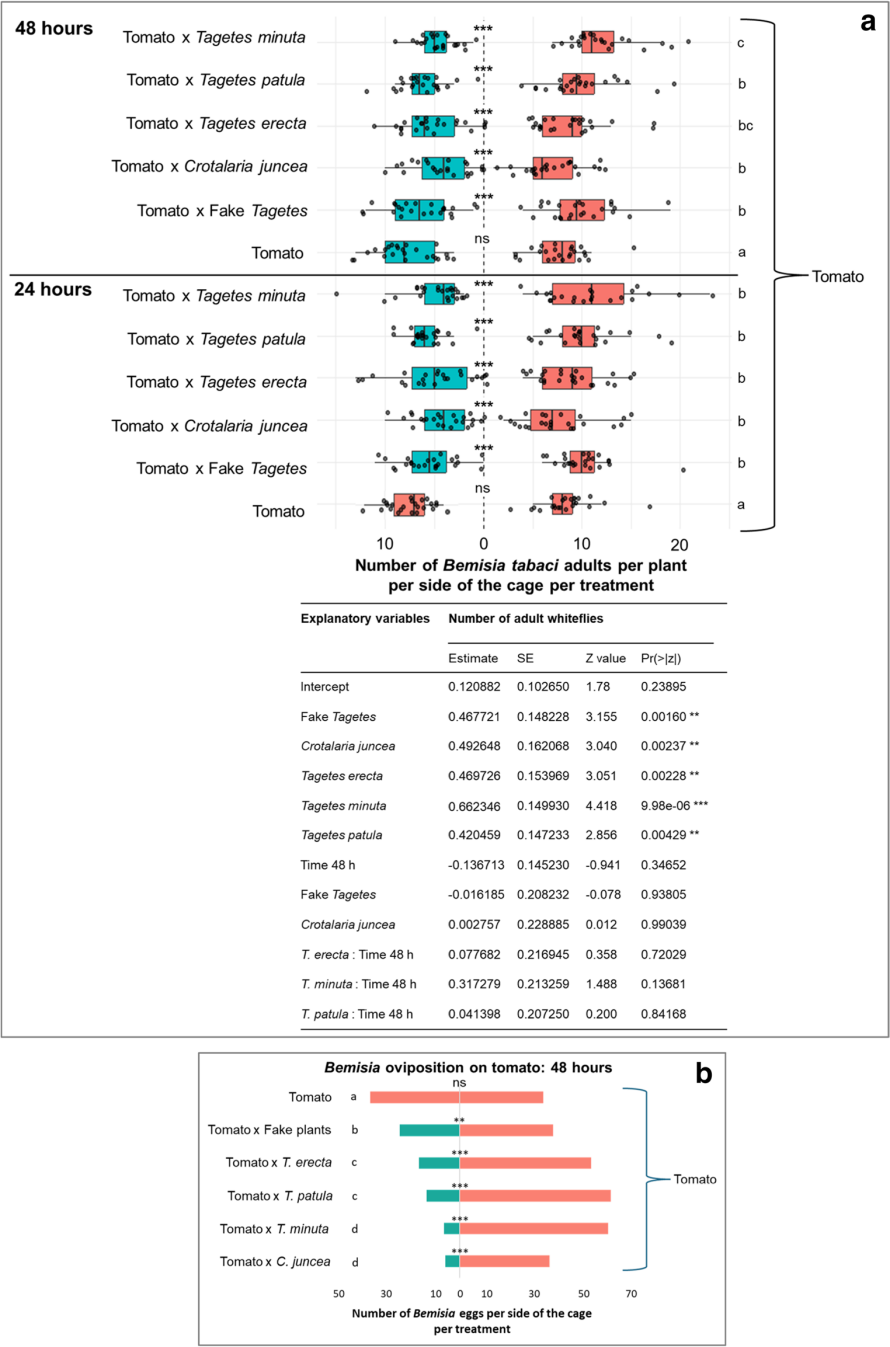
*Bemisia tabaci* adult settlement on tomato alone (red) vs. tomato associated with a biocontrol plant or a fake plant (turquoise) **in front** of it at 24 and 48 h **a**, and *B. tabaci* eggs oviposition at 48 h **b**. GLM binomial, Sidak, *P* < 0.05), *n* = 8 cages per free dual choice assay. Statistical results are given in the table below the figures

**Fig. 3:**
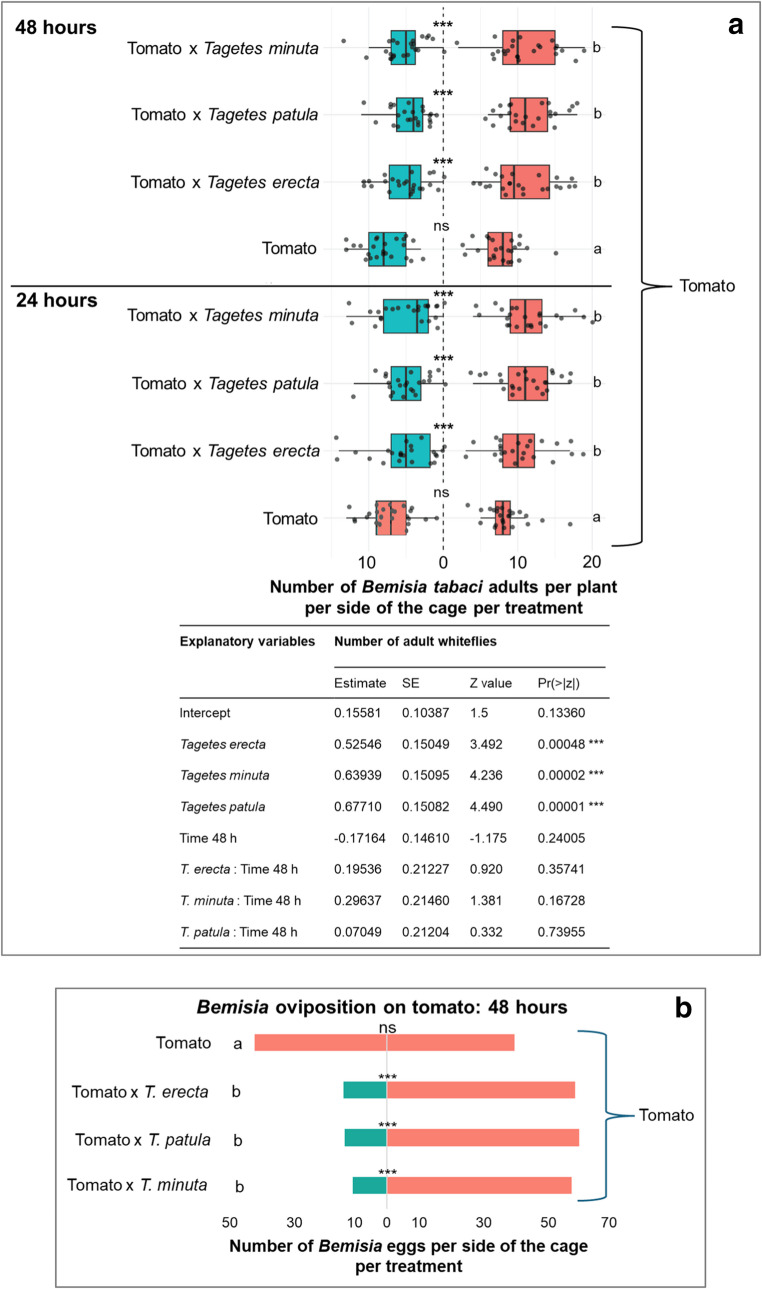
*Bemisia tabaci* adult settlement on tomato alone (red) vs. tomato associated with *Tagetes* **behind** (turquoise) at 24 and 48 h **a**, and *B. tabaci* eggs oviposition at 48 h **b**. GLM Binomial, Sidak, *P* < 0.05, *n* = 8 cages per free dual choice assay. Statistical results are given in the table below the figures

*Bemisia tabaci* adults settled (Figs. 2a and 3a) and oviposited (Figs. 2b and 3b) equally on either side of the cage with the tomato plants alone. A detailed analysis of oviposition sites revealed that egg-laying predominantly occurred on the lower parts of the tomato plants, followed by the middle parts (Fig. S1). The time variable at 24–48 h did not significantly affect whitefly behaviour (*P* > 0.05). These results validated the experimental setup of the free-dual choice assay for subsequent treatments in the climate chamber.

*Free-dual choice with biocontrol plants or fake plants* *in front* *-* The first setup of “tomato alone” on one side versus “tomato associated with a biocontrol plant in front” on the other. At 24 h, all *Tagetes* species (*T. erecta*, *T. patula*, and *T. minuta*) and *C. juncea* significantly reduced the number of *B. tabaci* adults that settled on tomato associated with a biocontrol plant compared to the control (tomato alone) (*P* < 0.01; Fig. 2a). The fake *Tagetes* plant also reduced the number of *B. tabaci* adults that settled on the side of the cage with tomato plants paired with it compared to the control side (*P* < 0.01; Fig. 2a).

Across the treatments at 24 h, any treatment with a companion plant (*T. erecta*,* T. patula*,* T. minuta*,* C. juncea*, or fake *Tagetes*) was different from the control tomato (*P* < 0.001; Fig. 2a). There were no significant differences detected among these biocontrol plant paired treatments themselves at this point, which were more biological effective in reducing the whiteflies than the fake *Tagetes* paired treatment.

Whitefly adult settlement at 48 h reinforced these trends. Tomatoes combined with *T. erecta*,* T. patula*,* T. minuta*,* C. juncea*, or fake *Tagetes* again displayed reduced adult numbers compared to the control (*P* < 0.01; Fig. 2a). Interestingly, *T. minuta* showed significantly lower settlement than *T. patula*,* C. juncea*, and fake *Tagetes*.

No significant differences were detected between 24 h and 48 h within treatment (i.e. time × treatment interaction non-significant, *P* > 0.05).

A similar trend was observed for the oviposited eggs at 48 h, which were significantly lower on the side of the tomato associated with a biocontrol plant (*P* < 0.001; Fig. 2b). Tomato paired with *C. juncea*, *T. minuta*, *T. patula*, and *T. erecta* showed the largest reduction in oviposition compared to the control, respectively. The fake plant also reduced oviposition, although less so than the biocontrol plants.

*Free-dual choice with biocontrol plants* *behind* *tomato -* The results at 24 h and 48 h were consistent with the first experiment, confirming that *Tagetes* species effectively repelled *B. tabaci* (Fig. 3). This led to lower adult settlement (Fig. 3a) and oviposition (Fig. 3b) on tomato plants associated with the *Tagetes* than on the tomato control (*P* < 0.001). The repellent effect of the three *Tagetes* species treatments was statistically similar both at 24 h and 48 h; so was the oviposition at 48 h.

Again, no significant differences between 24 h and 48 h were detected.

In both setups, we observed barely any *B. tabaci* adults directly on the *Tagetes* species, unlike on *Crotalaria*. *Tagetes erecta*,* T. patula*, and *T. minuta* had no *B. tabaci* per plant at 24-hour sampling and 0–2 per plant at 48 h. *Crotalaria* had 11–25 *B. tabaci* per plant at 24 h and 48 h.

### Tagetes VOCs Analysis

The relative abundance of VOCs emitted by the different species of *Tagetes* and the fake plants is detailed in Table S1 (Suppl. Data. See also chromatogramms Fig. S3). A Total of 26 compounds were identified in all samples. The compounds emitted by *Tagetes* spp. can be grouped into two chemical functions and two families of compounds, respectively: ketones, aldehydes, oxygenated monoterpenes, and hydrocarbon monoterpenes. Moreover, with fake plants, there were also aromatic hydrocarbons. Twelve compounds, representing between 5% and 13% of the total area, depending on the species, were detected in *Tagetes* species but were unidentified. For further analyses, compounds found in the blank control were not included: nonanal, decanal, toluene, phenol, and 5-Hepten-2-one, 6-methyl.

**Fig. 4:**
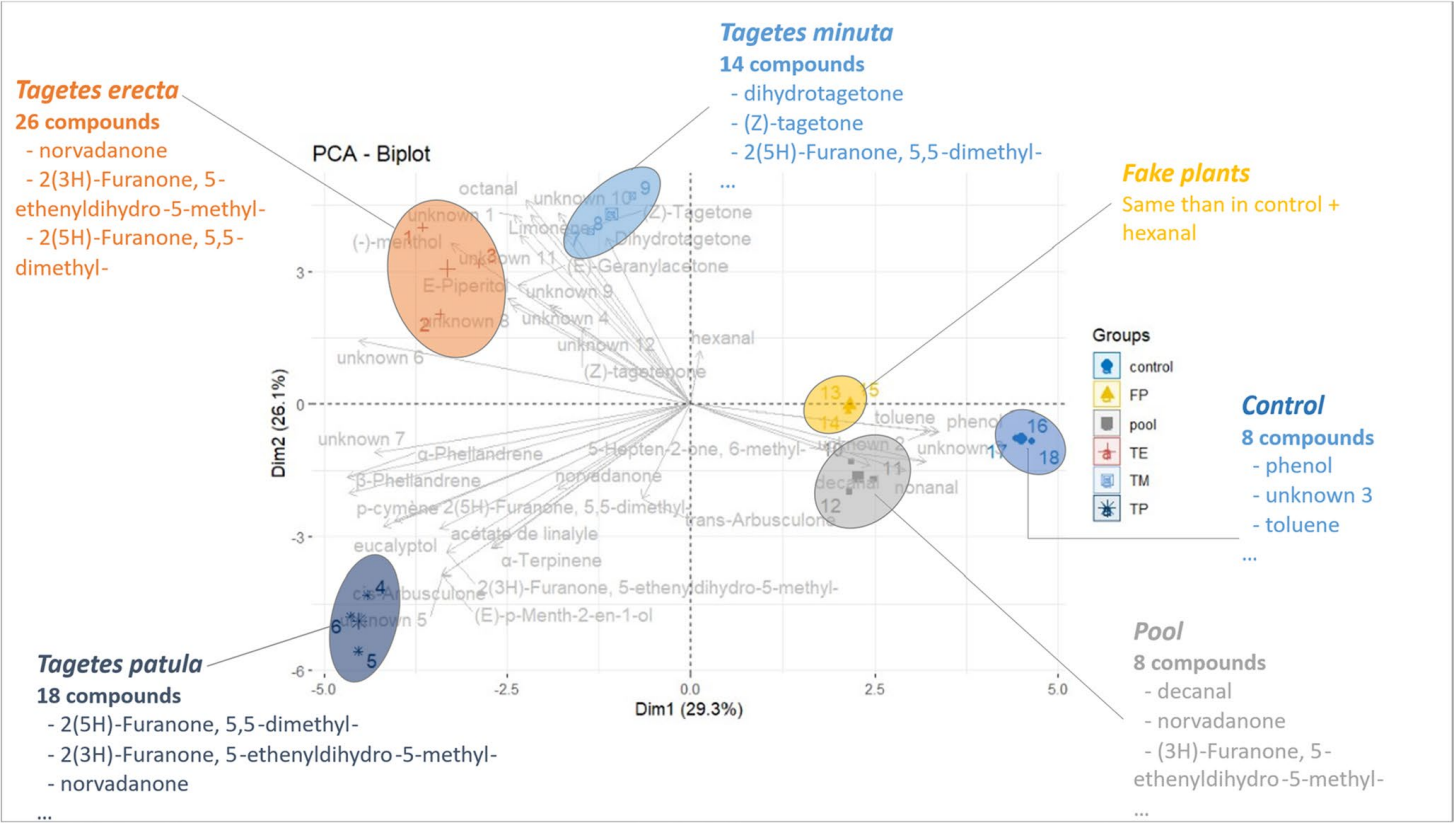
Principal component analysis (PCA): VOC blend composition for the three *Tagetes* species, the pool, the control, and the fake plants. Contamination was removed from the *Tagetes* sampling. For each area, there are three replicates and the average

Volatile organic compounds composition clearly distinguishes the array of VOCs emitted by the three different *Tagetes* species as well as the pool and the fake plants (Fig. 4). Despite *Tagetes* specificities, ketones, with typical compounds such as the tagetone were the most important class of compounds emitted by *T. erecta* (57.9 ± 22.1%), *T. patula* (72.13 ± 5.64%) and *T. minuta* (65.41 ± 25.59%).

**Fig. 5:**
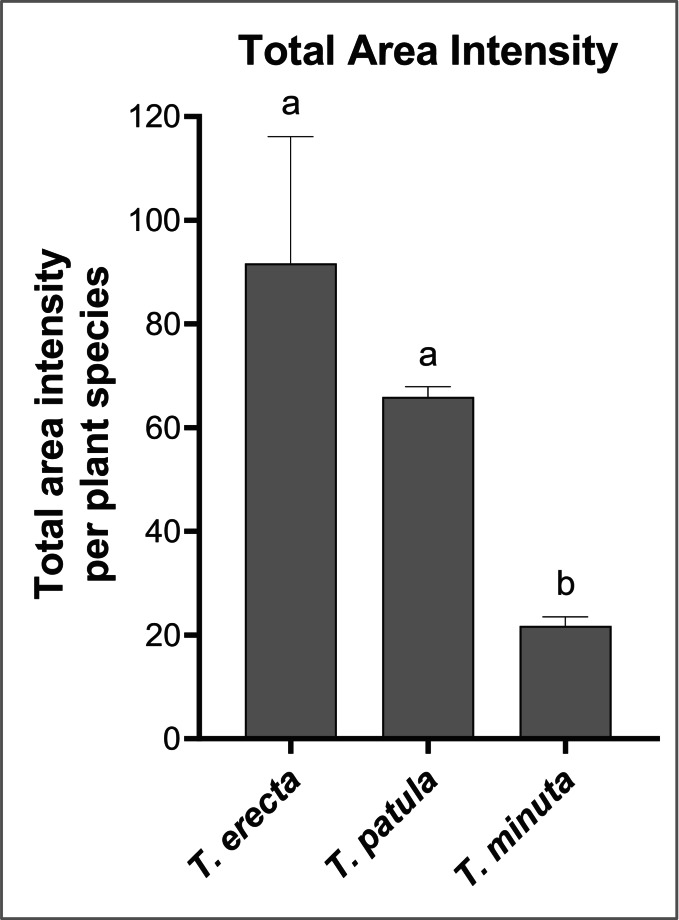
Total area intensity for *Tagetes erecta*, *T. patula*, and *T. minuta* (GLM Gamma, Tukey post hoc, *P* **<** 0.05)

*Tagetes minuta* emitted fewer compounds (14) and in the smallest quantity, mainly ketones (Fig. 5 & chromatogram Suppl. Data, Fig. S3a). The main compounds detected were dihydrotagetone, (*Z*)-Tagetone, 2(5 H)-Furanone, 5,5-dimethyl-, and (*E*)-Geranylacetone.

*Tagetes patula* showed an intermediate number of compounds (18), emitted in an intermediate quantity but statistically similar *T. erecta* (Fig. 5 & chromatogram Suppl. Data, Fig. S3b). The main compounds found were 2(5 H)-Furanone, 5,5-dimethyl-, the 2(3 H)-Furanone, 5-ethenyldihydro-5-methyl-, and norvadanone. There were eight monoterpenes and four compounds specifically emitted by this species: cis-arbusculone, α-terpinene, (*E*)-p-Menth-2-en-1-ol, and the unknown 5.

*Tagetes erecta* emitted the highest number of compounds (26) in the highest quantity (Fig. 5 & chromatogram Suppl. Data, Fig. S3c). The species emitted all types of compounds identified. The main compounds are similar to *T. patula*, plus the E-geranylacetone and the Z-tagetone, shared with *T. minuta*. Z-tagetenone is specific to *T. erecta*, but it might also be because it is emitted in the highest quantity, making it detectable only in this high-emitter species. Five other compounds are also specific to *T. erecta*: E-piperitol, and unknown 4, 8, 9, and 12.

The pool samples represented the compounds present in the atmosphere of a greenhouse compartment containing the *Tagetes* plants from the three species. Interestingly, it contained compounds from the greenhouse compartment (shared with control samples) and some of the major compounds emitted by the *Tagetes* plants, including nordavanone, furanones, trans-arbusculone, and E-geranylacetone. The air in the greenhouse compartment is therefore loaded with aromatic molecules from the plants.

The fake plant samples contained similar compounds to the control ones, i.e. the compounds present in the atmosphere of the empty greenhouse compartment, highlighting the relevance of their use for demonstrating the physical barrier effect, as they don’t seem to emit any specific chemical blend, at least that could be trapped with our sampling setup.

## Discussion

The whiteflies (*Bemisia tabaci*) were distributed evenly between tomato plants on the left and right sides of the cages in the control treatments, with no significant differences in adult settlement or oviposition. This even distribution validated the robustness of the experimental setup in the growth chamber. It confirmed that whitefly dispersion within the cages was not influenced by external environmental factors but rather reflected their natural feeding and oviposition behaviour. Establishing the reliability of the setup was essential to ensure that any differences observed in the presence of biocontrol plants or fake plants could be confidently attributed to the effects of the treatments.

This study provides evidence that the integration of biocontrol plants, particularly *Tagetes* species and *C. juncea*, can significantly reduce whitefly adult settlement and oviposition on tomato plants under controlled conditions. The results allow a fine dissection of the chemical versus physical mechanisms underlying pest deterrence, thanks to the inclusion of a synthetic, odourless fake *Tagetes* as a physical barrier control.

All *Tagetes* species deterred whiteflies to a similar extent. This finding is consistent with literature reports of specific *Tagetes* species deterring certain whitefly species. However, this is the first time that the deterrent effects of the three *Tagetes* species are directly compared. *Tagetes erecta* was able to reduce the incidence of unspecified whitefly species and their viral transmission in a tomato field (Zavaleta-Mejía and Gómez 1995). *Tagetes patula* was shown to be repellent to the whitefly, *Trialeurodes vaporariorum* (Conboy et al. 2019). Some *Tagetes minuta*’s volatiles are attractive to *T. vaporariorum* (Matu et al. 2020) and repellent to thrips (*Megalurothrips sjostedti* females, Diabate et al. 2019). However, to the best of our knowledge, this is the first time that a repellent effect of *T. minuta* on *B. tabaci* is demonstrated.

The deterrent effect of the three *Tagetes* species on whiteflies could be linked to two different modes of action: *Tagetes* can act as a physical barrier, blocking the flying dispersion of the insect, or they can act as a chemical repellent linked to a particular release of volatile molecules (Parker et al. 2013; Djian-Caporalino and Lavoir 2024). Our experimental setup allowed us to test the effectiveness of each mode of action. Adding fake plants mimicking biocontrol plants in front of the tomato plants allowed us to test the physical barrier effect without chemical release. Indeed, the chemical analysis of the fake plant blend concluded to a neutral material as only hexanal was sampled in addition to the compounds identified in the blank, i.e. in the air of the compartment. Our results confirmed a physical barrier effect from the *Tagetes* species, as the fake plants mimic the biomass of the three *Tagetes* species. Although our assays were conducted in cages with plants of the same size, in the greenhouse, the three species generally exhibit similar stature (height and density), as they are all giant varieties. This means they should have more/less the same physical barrier effect, except perhaps in cases where dwarf varieties are used. Plants with tall or large canopies usually serve well as mechanical barriers (Fereres 2000; Djian-Caporalino and Lavoir 2024).

The *Tagetes* species are also repellent due to VOCs, as confirmed by our second setup with *Tagetes* behind the tomato, as was with Conboy et al. (2019), but on another whitefly species. Also, there were hardly any whiteflies observed on the *Tagetes* plants during our samplings. Interestingly, the three *Tagetes* species repel whiteflies with similar intensity, even though they showed distinct blends. For instance, the repellent effect did not seem linked to the intensity of the blend, as *T. minuta* deterred more or similarly whiteflies than *T. erecta* and despite a 10-fold difference in intensity.

The GC-MS analysis identified known repellent VOCs in all three *Tagetes* species. Previously, *T. patula* was shown to have an insecticidal/repellent effect attributed to limonene on *B. tabaci* (Fabrick et al. 2020) and *T. vaporariorum* (Conboy et al. 2019). Also, (*Z*)-β-ocimene elicited strong repellence in the low dosages of 0.1% and 1% to *T. vaporariorum* (Matu et al. 2020). In our study, limonene was identified in the blend of the three species, but in small quantities. However, ketones should be tested as well to check for their potential repellent effects.

On the contrary, olfactometer assays found that flowering and vegetative *T. minuta* were preferred over tomato by another whitefly (*T. vaporariorum*) and related attractive compounds ((*Z*)−3-hexenyl acetate) were found in this marigold (Matu et al. 2020). The chemical blend effect of marigolds on whiteflies could then depend on the whitefly species but also on the phenological stage of the plant, as for nematodes (Njekete 2025).

*Crotalaria juncea* was also confirmed, as anticipated, to function as a deterrent in the “biocontrol in front” setup by limiting whitefly settlement and oviposition on tomato. Unexpectedly, however, *C. juncea* attracted significantly more adult whiteflies and oviposited eggs than the tomato paired with it, indicating a potential trapping effect. This finding deviated from the initial hypothesis, which assumed a solely mechanical barrier function. The pronounced attraction raises questions about the role of unidentified chemical cues or substrate-related factors that might influence *B. tabaci* preference. Moreover, it remains unknown whether a similar attraction would be observed if *C. juncea* were positioned behind the tomato, as was done for the *Tagetes* species. These results suggest that *C. juncea* operates via a different mode of action compared to *Tagetes*, potentially acting as a sink or reservoir for *B. tabaci* adults.

Notably, more whiteflies and eggs were recorded on *C. juncea* than on the adjacent tomato, pointing to a level of attractiveness that could equal or even exceed that of the host crop. Previous studies have shown that *C. juncea* can reduce *B. tabaci* incidence and its associated disorders, such as squash silverleaf, primarily through physical barrier effects (Manandhar et al. 2009; Deberdt and Fernandes 2013). However, while the high egg deposition suggests effective attraction, it is not yet known whether this results in successful whitefly development—an important criterion for classifying *C. juncea* as a dead-end trap plant rather than merely a trap plant. Interestingly, *C. juncea* has already demonstrated dead-end trap plant characteristics against root-knot nematodes, allowing juvenile penetration while suppressing reproduction and development (Njekete et al. 2025). Whether similar mechanisms apply to whitefly interactions with *C. juncea* remains to be determined and deserves further study.

## Conclusion and Perspectives

This study demonstrates that *Tagetes* species (notably *T. erecta*, *T. patula*, and *T. minuta*) and *Crotalaria juncea* are effective in deterring *Bemisia tabaci* from tomato plants through distinct, yet complementary, mechanisms. *Tagetes* acted primarily through repellence and potential physical barrier effects, while *Crotalaria* functioned as a trap plant, attracting and sequestering adult whiteflies.

The physical barrier effect, particularly in *Tagetes* and *Crotalaria*, appears to correlate with morphological features such as plant height, architecture, and aerial biomass. Species with similar structures are likely to exert comparable physical interference with pest movement. However, when it comes to chemical repellence, the story is different. The repellence is linked to one compound, a mixture of compounds, or other compounds in nature and intensity, all of which can have the same effect. This seems to be the case with different *Tagete*s species, as the three species, with quantitatively and qualitatively different blends, repelled the pest similarly. This could indicate the possibility of several chemical strategies that are effective in repelling the pest. However, other criteria—such as how *Tagetes* interacts with the crop, its potential to target multiple pests, promotion of other ecosystem services, seed availability, and ease of agronomic handling—could also influence the choice of *Tagetes* species (Njekete 2025; Moreau et al. 2025). Further investigation is required to identify the active molecules responsible, to assess their dose-dependent activity, and to explore their potential for breeding or isolation for biopesticide development. Likewise, the impact of plant phenology must be clarified—particularly whether repellence diminishes after flowering, which could complicate practical field deployment.

*Crotalaria juncea* offered a contrasting mode of action, operating as a potential trap plant. It attracted significantly more whiteflies and eggs than tomato, which may suggest chemical or substrate-based cues. While the physical barrier effect of *C. juncea* was anticipated, the unexpected attraction raises important research questions: does *C. juncea* emit attractive volatiles, and if so, are they consistent across growing conditions? More importantly, can *C. juncea* serve as a dead-end trap plant? If confirmed, such a role would significantly strengthen its use in multi-pest management. However, when not a dead-end trap, trap cropping presents logistical and ecological challenges. Before field deployment, it is essential to evaluate pest preference between the crop and trap plant, ensure spatial arrangement enhances the sink effect, and determine whether the trap plant must be destroyed after pest settlement to prevent it from becoming a pest reservoir.

In summary, these findings confirm the potential of biocontrol plants such as *Tagetes* and *Crotalaria* in reducing pest pressure, thereby contributing to more sustainable tomato production systems. Their effects may extend beyond deterrence, potentially influencing pest reproduction and survival. On-plant assays of *T. patula* reduced *B. tabaci* survival more than on bean (Fabrick et al. 2020) while VOCs emitted by flowers of *T. patula* significantly reduced aphid reproduction (Dardouri et al. 2017), implying both antibiosis and antixenosis mechanisms (Stenberg and Muola 2017). Furthermore, these plants offer multiple ecosystem services beyond pest suppression—including pollinator support, soil improvement, and weed management—further validating their place as multi-service plants in diversified agrosystems (Letourneau et al. 2011; Balzan 2017; Gardarin et al. 2022; Djian-Caporalino et al. 2024). Future research should focus on validating the repellent activity of specific volatile compounds identified in *Tagetes* spp. through targeted bioassays with isolated VOCs. It is also essential to investigate cultivar-specific effects and assess the long-term consistency of deterrence under semi-field and open-field conditions. In the case of *C. juncea*, the unexpected attraction of whiteflies warrants further chemical analysis to determine whether attractant volatiles are involved. Understanding whether *B. tabaci* completes its life cycle on *C. juncea* is vital to confirming its status as a dead-end trap plant. Combining *Tagetes* and *Crotalaria* in a push–pull system could offer complementary control effects and should be explored further under diverse cropping conditions.

## Supplementary Information

Below is the link to the electronic supplementary material.

ESM 1 (DOCX 767 KB)

## Data Availability

No datasets were generated or analysed during the current study.
