# Supplementary material for: Altered Behavioural Response of Whitefly (Bemisia tabaci) on Tomato Associated with Biocontrol Plants
Source: J Chem Ecol. 2025 Oct 6;51(5):98. doi: 10.1007/s10886-025-01649-4 (PMC12500831; doi:10.1007/s10886-025-01649-4)
Supplement: Supplementary file 1 — (DOCX 767 KB) [file 10886_2025_1649_MOESM1_ESM.docx]

**Supplementary material**

**ALTERED BEHAVIOURAL RESPONSE OF WHITEFLY (*Bemisia tabaci*) ON TOMATO ASSOCIATED WITH BIOCONTROL PLANTS**

CLIVEN NJEKETE, ALBANE NOEL, SAMUEL MATSINHE, XAVIER FERNANDEZ, CAROLINE DJIAN-CAPORALINO, ANNE-VIOLETTE LAVOIR

The plant’s location was important for the behaviour of the whitefly as they preferred to lay eggs on the bottom part of the tomato plant, followed by the middle, and then the top (Fig. S1). Arnó et al. (2006) found *B. tabaci* eggs mostly in the middle stratum, whereas the nymphs mainly clustered in the bottom stratum of tomato (Arnó et al. 2006). Insect distribution on the plant strata, which is influenced by plant physiology, biochemistry, structure, level of environmental heterogeneity, specific behaviour, and movement patterns of the insect, is important in insect monitoring and sampling (Ekbom and Xu 1990).


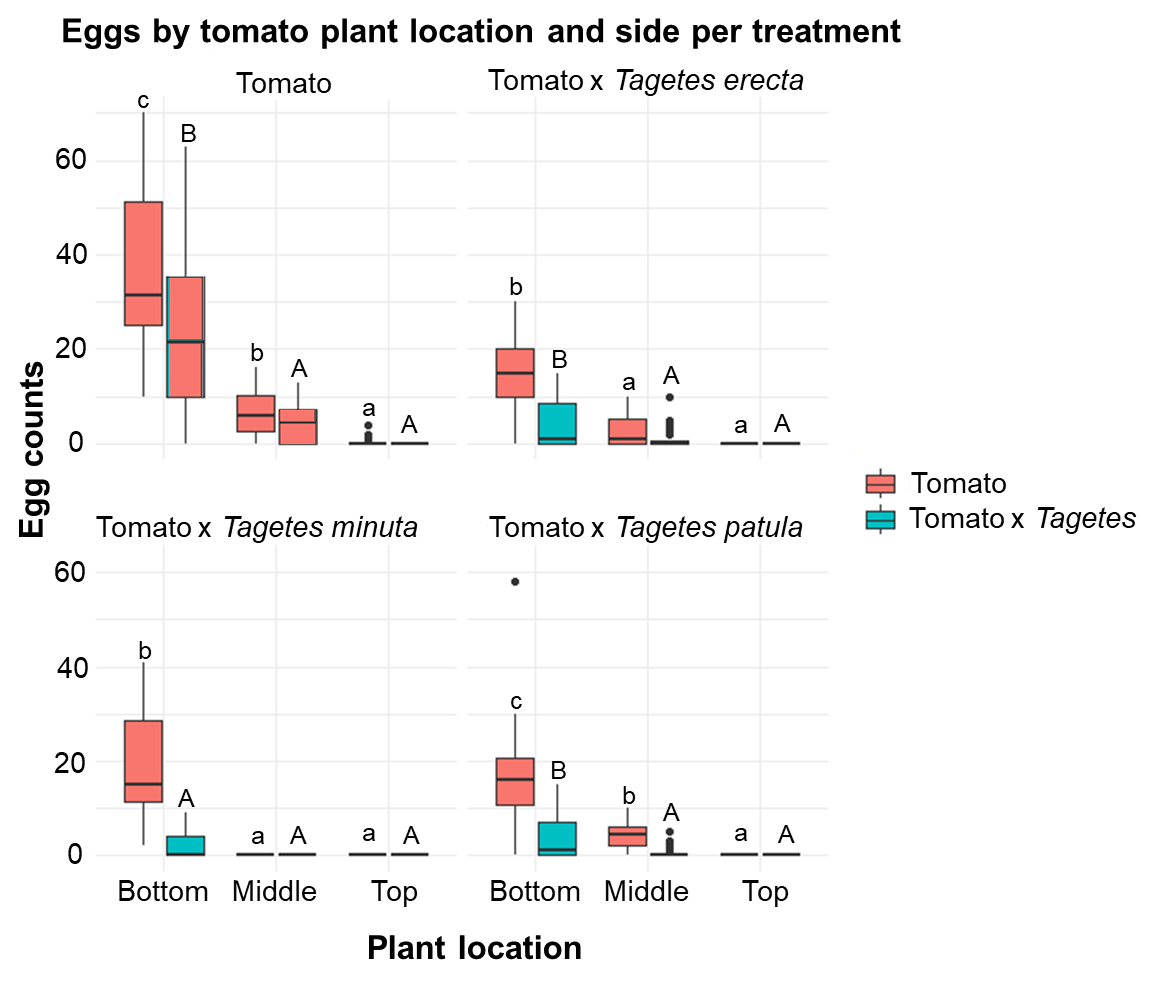


**Fig. S1** The number of eggs oviposited by tomato plant location and side per treatment for the tomato alone (red) and tomato with a *Tagetes* species (turquoise). GLM Binomial, Tukey, *P* < 0.05, n = 8 cages per free dual choice assay


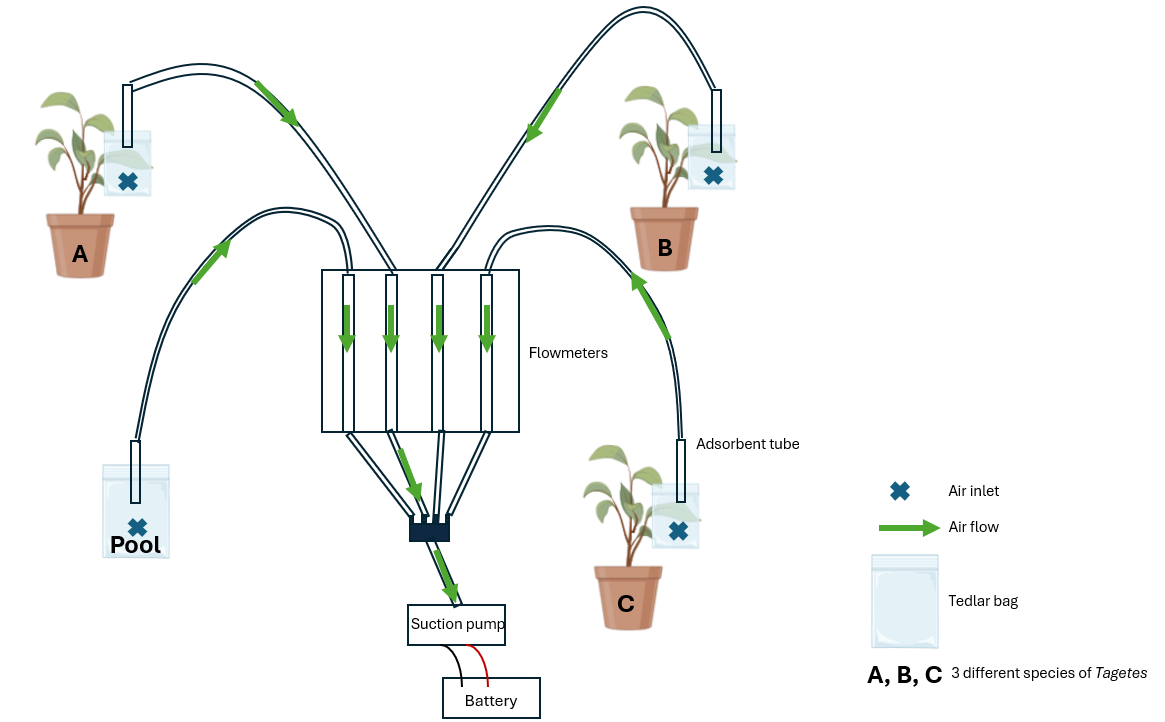


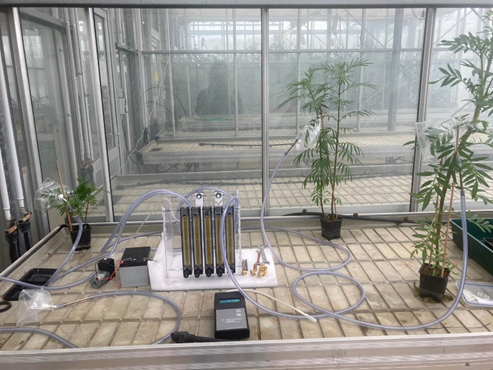


**Fig. S2** Sampling design for volatile organic compounds


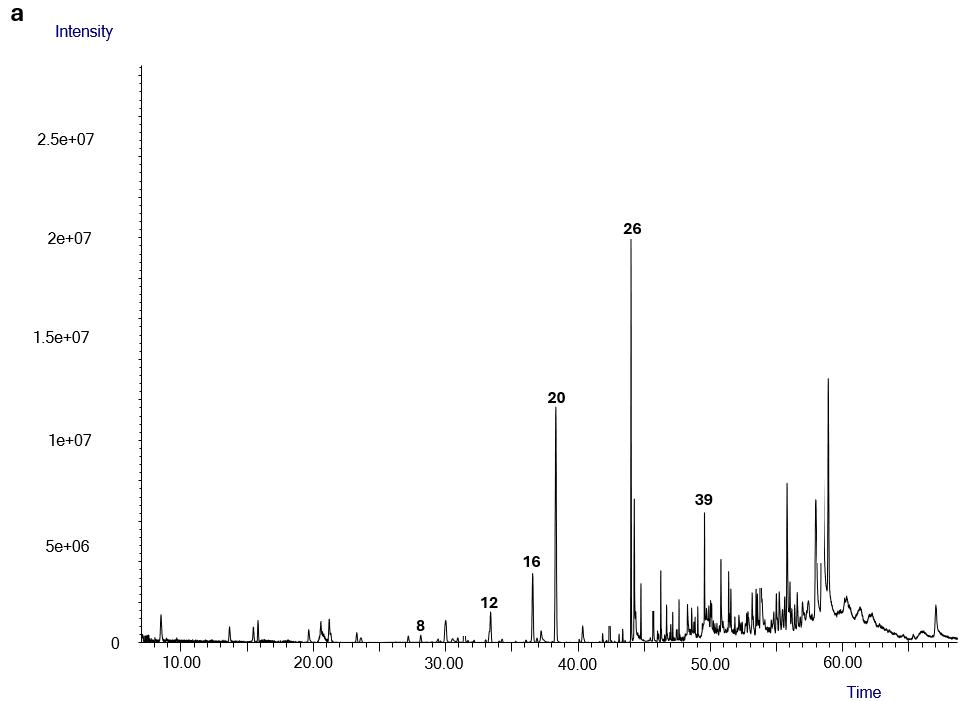


***Tagetes minuta***


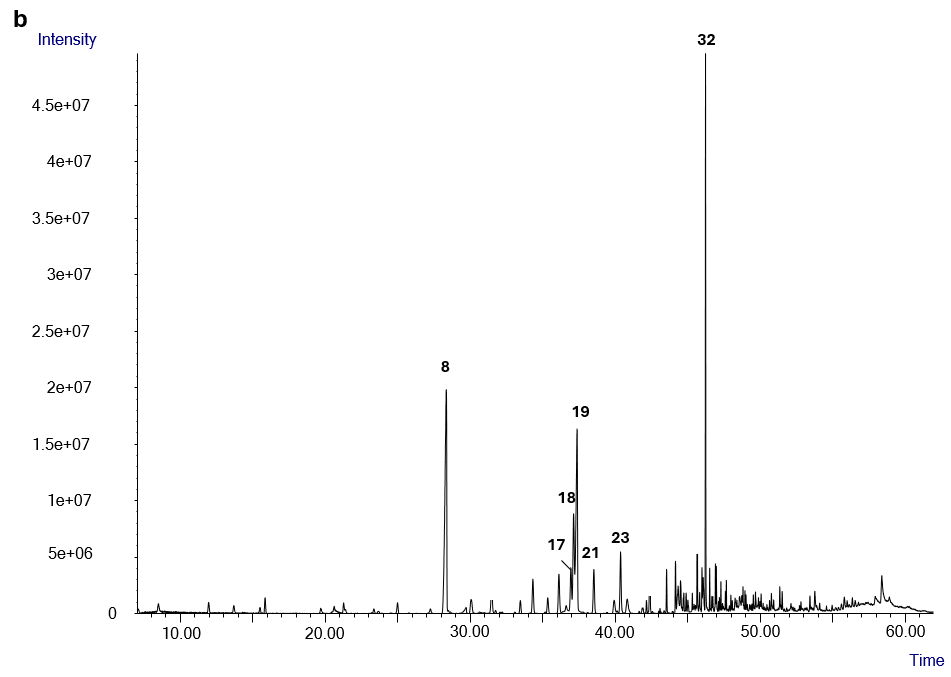


***Tagetes patula***


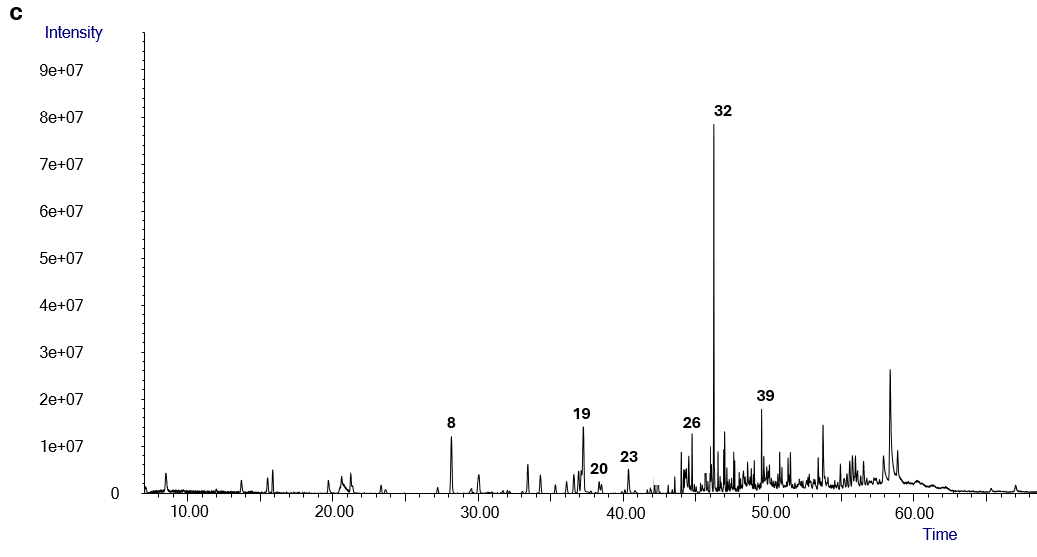


***Tagetes erecta***


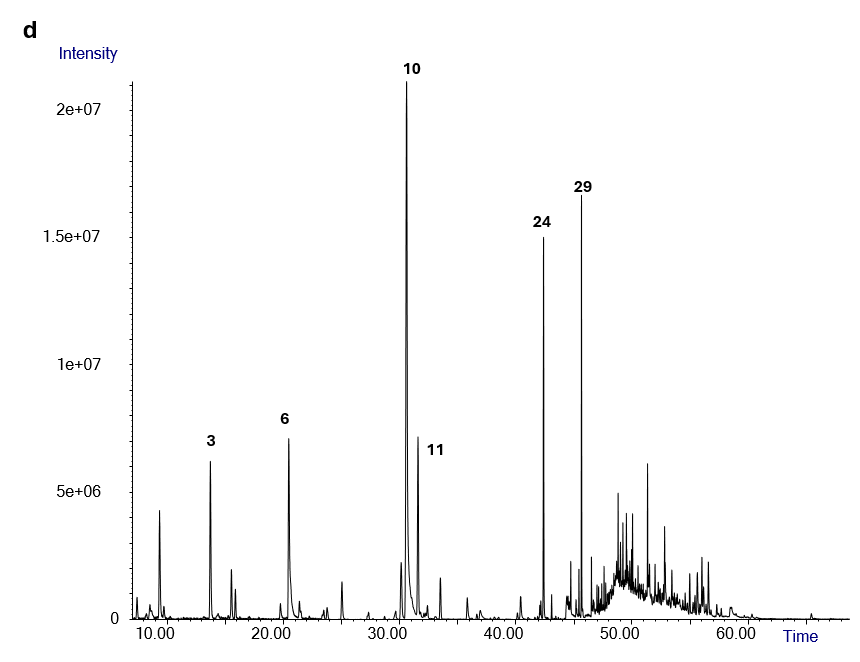

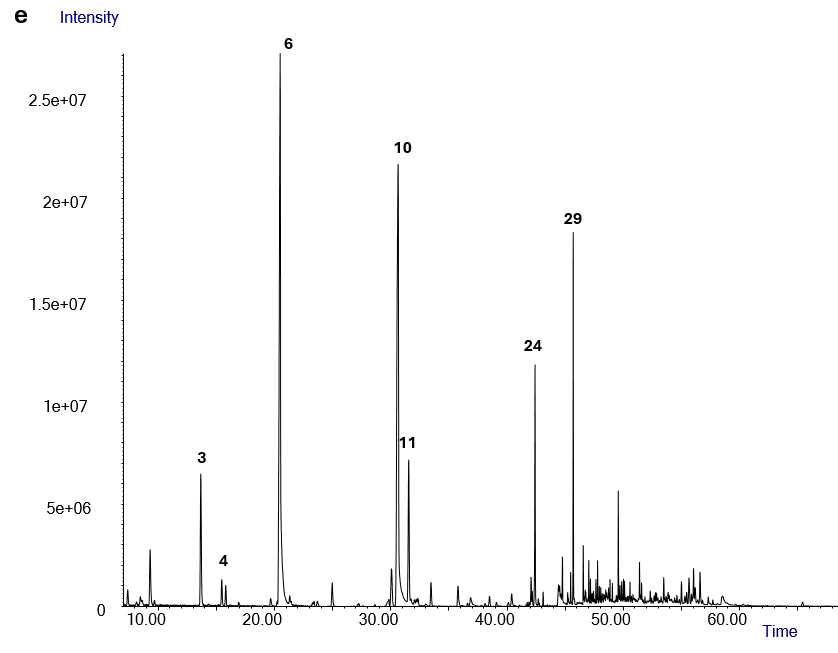


**Fake plant**

**Blank control**


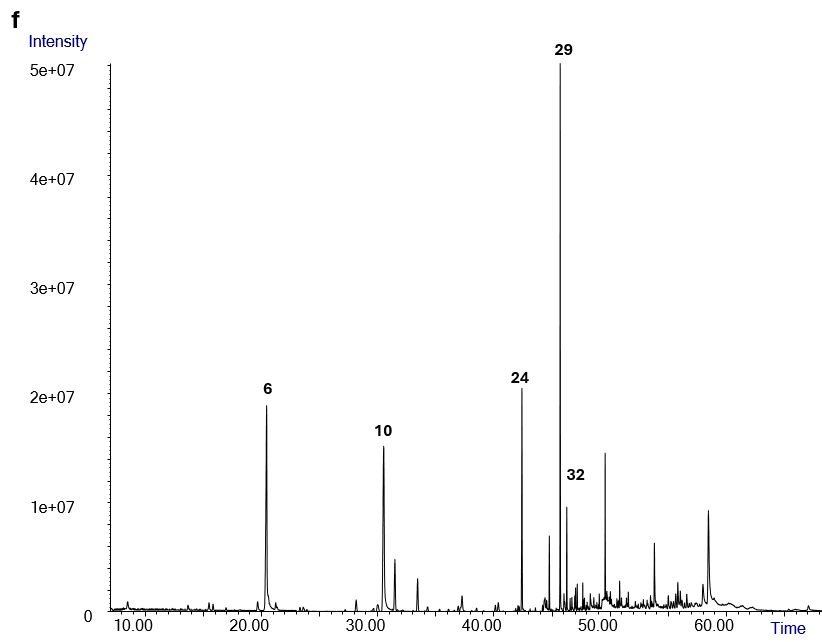


**Pool sample**

**Fig. S3** Total ion chromatograms (TICs) of compounds detected by DHS ATD-GC-MS analysis on (a) *Tagetes minuta*, (b) *Tagetes patula*, (c) *Tagetes erecta*, (d) fake plant, (e) blank control, (f) the pool sample. The numbers correspond to the compounds detailed in Table S1. Contaminants for *Tagetes* species are not represented

**Table S1** Relative percentages of *Tagetes erecta* (TE), *Tagetes patula* (TP), *Tagetes minuta* (TM), fake plants (FP), pool samples and controls by DHS ATD-GC-MS.

|  |  |  |  |  |  |  | Area (% ± SD) ^(a)^ | | | | | |
| --- | --- | --- | --- | --- | --- | --- | --- | --- | --- | --- | --- | --- |
| Number | Compound | Family | Retention time (min) | RI (calc.) | RI (th.) | CAS number | TE | TP | TM | FP | Pool | Control |
| 4 | hexanal | Aldehyde | 15.48 | 801 | 801 | 66-25-1 | 1.12±1.03 | - | - | 1.49±1.35 | - | - |
| 12 | octanal | Aldehyde | 33.4 | 1003 | 1003 | 124-13-0 | 3.08±1.16 | - | 5.12±0.41 | - | - | - |
| 24 | nonanal^(b)^ | Aldehyde | 42.37 | 1106 | 1104 | 124-19-6 | * | * | * | **10.29±0.57** | 17.51±4.48 | 6.44±0.54 |
| 29 | decanal^(b)^ | Aldehyde | 45.66 | 1208 | 1206 | 112-31-2 | * | * | * | 6.7±0.94 | 25.44±9.38 | 5.92±1.22 |
| 3 | toluene^(b)^ | Aromatic hydrocarbon | 13.71 | 771 | 763 | 108-88-3 | - | - | - | 8.77±1.71 | - | 7.76±2.62 |
| 10 | phenol^(b)^ | Aromatic hydrocarbon | 36.63 | 974 | 981 | 108-95-2 | - | - | - | 43.63±4.76 | - | 33.83±2.24 |
| 8 | 2(5H)-Furanone, 5,5-dimethyl- | Ketone | 28.14 | 948 | 954 | 20019-64-1 | **8.21±2.09** | **26.52±0.76** | **12.16±3.93** | - | 5.48±4.89 | - |
| 11 | 5-Hepten-2-one, 6-methyl-^(b)^ | Ketone | 31.43 | 984 | 986 | 110-93-0 | * | * | * | 6.45±2.37 | 7.05±6.16 | 6.21±1.62 |
| 19 | 2(3H)-Furanone, 5-ethenyldihydro-5-methyl- | Ketone | 37.24 | 1045 | 1043 | 1073-11-6 | **12.73±5.80** | **18.50±0.40** | - | - | 10.97±2.10 | - |
| 20 | Dihydrotagetone | Ketone | 38.31 | 1057 | 1047 | 1879-00-1 | 3.32±0.63 | - | **23.87±9.04** | - | - | - |
| 21 | cis-Arbusculone | Ketone | 38.51 | 1059 | 1052 | 56469-37-5 | - | 3.52±0.15 | - | - | - | - |
| 23 | trans-Arbusculone | Ketone | 40.36 | 1080 | 1071 | 56469-36-4 | 3.76±1.04 | **4.59±0.21** | 1.38±2.39 | - | 8.79±0.91 | - |
| 26 | (Z)-Tagetone | Ketone | 44.23 | 1155 | 1155 | 3588-18-9 | **4.38±1.81** | 0.61±1.06 | **14.05±7.49** | - | - | - |
| 32 | nordavanone | Ketone | 46.23 | 1237 | 1230 | 54933-91-4 | **19.07±6.04** | **15.39±1.27** | 3.81±0.63 | - | 22.36±4.15 | - |
| 33 | (Z)-tagetenone | Ketone | 46.28 | 1239 | 1231 | 33746-71-3 | 0.69±1.19 | - | - | - | - | - |
| 39 | (E)-Geranylacetone | Ketone | 49.53 | 1452 | 1453 | 3796-70-1 | **5.74±3.61** | 3.03±1.79 | **10.14±2.11** | - | 2.4±4.16 | - |
| 13 | α-Phellandrene | Monoterpene hydrocarbon | 34.27 | 1012 | 1005 | 99-83-2 | 2.67±0.60 | 2.63±0.26 | - | - | - | - |
| 14 | α-Terpinene | Monoterpene hydrocarbon | 35.33 | 1024 | 1017 | 99-86-5 | - | 0.85±0.74 | - | - | - | - |
| 15 | p-cymene | Monoterpene hydrocarbon | 36.07 | 1032 | 1025 | 99-87-6 | 2.56±3.09 | 3.50±0.21 | - | - | - | - |
| 16 | Limonene | Monoterpene hydrocarbon | 36.58 | 1038 | 1030 | 138-86-3 | 3.20±0.80 | 0.35±0.61 | **7.81±1.68** | - | - | - |
| 17 | β-Phellandrene | Monoterpene hydrocarbon | 36.9 | 1041 | 1031 | 555-10-2 | 3.13±0.76 | 3.61±0.37 | - | - | - | - |
| 25 | (E)-p-Menth-2-en-1-ol | Monoterpene hydrocarbon | 43.54 | 1141 | 1141 | 29803-81-4 | - | 1.70±0.11 | - | - | - | - |
| 18 | eucalyptol | Oxygenated monoterpene | 37.12 | 1044 | 1032 | 470-82-6 | - | **8.43±1.15** | 3.60±6.23 | - | - | - |
| 28 | (-)-menthol | Oxygenated monoterpene | 44.74 | 1177 | 1175 | 2216-51-5 | 2.72±1.03 | 0.63±1.09 | 3.13±0.22 | - | - | - |
| 31 | E-Piperitol | Oxygenated monoterpene | 46.05 | 1228 | 1208 | 16721-39-4 | 3.26±1.14 | - | - | - | - | - |
| 1 | unknown 1 |  | 8.47 | 688 | - |  | 3.61±2.08 | - | 4.11±1.19 | - | - | - |
| 2 | unknown 2^(b)^ |  | 9.33 | 697 | - |  | - | - | - |  |  | 2.74±0.86 |
| 6 | unknown 3^(b)^ |  | 20.49 | 863 | - | - | - | - | - | 19.16±5.51 | - | 34.66±3.92 |
| 7 | unknown 4 |  | 21.21 | 872 | - | - | 1.35±1.33 | - | - | - | - | - |
| 22 | unknown 5 |  | 39.88 | 1075 | - | - | - | 1.42±0.03 | - | - | - | - |
| 27 | unknown 6 |  | 44.34 | 1165 | - | - | 2.93±1.55 | 2.57±0.92 | 4.34±1.04 | - | - | - |
| 30 | unknown 7 |  | 45.99 | 1225 | - | - | 2.63±0.89 | 2.15±1.16 | - | - | - | - |
| 34 | unknown 8 |  | 46.92 | 1273 | - | - | 2.53±1.36 | - | - | - | - | - |
| 35 | unknown 9 |  | 46.98 | 1276 | - | - | 1.75±1.62 | - | - | - | - | - |
| 36 | unknown 10 |  | 48.28 | 1357 | - | - | 1.19±1.06 | - | 3.52±0.96 | - | - | - |
| 37 | unknown 11 |  | 48.56 | 1377 | - | - | 2.60±0.87 | - | 2.97±2.60 | - | - | - |
| 38 | unknown 12 |  | 49.02 | 1410 | - | - | 0.46±0.80 | - | - | - | - | - |
| 5 | degradation column compound |  | 15.83 | 806 | - | - | 1.33±1.23 | - | - | - | - | - |
| 9 | degradation column compound^(b)^ |  | 30.01 | 968 | - | - | * | * | * | 3.51±0.50 | - | 2.45±0.28 |

^(a)^Area expressed as a percentage of the total area of compounds detected with relative percentages <1%
^(b)^ Compounds present in the control sample
SD, standard deviation
RI (cal.), Retention index calculated according to retention times of standard n-alkanes C6-C18 mixture.
 RI (th.), Retention index reported in the literature (NIST)
 -, absent.
*, present but not counted in the % of the total area for the 3 *Tagetes* species
 **7.3±0.93**, 5 major compounds for each species (without compounds in the control)
